# Overcoming barriers to gut microbiome development through nutritional factors in the first 1,000 days of life: strategies and implications for preventing non-communicable diseases

**DOI:** 10.1017/gmb.2025.10014

**Published:** 2025-09-10

**Authors:** Zoe Teh, Cristina Garcia-Maurino Alcazar, Komal Bhatia

**Affiliations:** 1 Institute for Global Health, University College London, London, UK; 2 ISGlobal, Barcelona Institute for Global Health, Barcelona, Spain

**Keywords:** microbiome, non-communicable disease, infant and child nutrition, maternal nutrition, breastfeeding

## Abstract

Current efforts to reduce the incidence of non-communicable disease (NCD) are slow, but increasing evidence highlights the microbiome as a potential target for prevention. The majority of microbial development occurs in the first 1,000 days of life, presenting opportunities for strategic intervention to reduce the prevalence of future NCDs. In this review, we explore the social, structural, and political barriers that may hinder physiological gut microbial development in the first 1,000 days in the context of current scientific knowledge, focusing on nutritional factors in pregnancy, and during the exclusive breastfeeding and complementary feeding periods. We summarise emerging evidence and explore obstacles to nutritional choices affecting microbial development, and unpack the rhetoric that healthy eating to develop a microbiome that supports optimum health is an individual choice. As evidence on the role of the microbiome in health and disease grows, specific attention must be applied to existing social, structural, and political barriers that may hinder optimal microbial development. Addressing the role of corporate actors and social determinants influencing dietary choices and barriers surrounding breastfeeding must be prioritised, alongside efforts to advance basic scientific research. Until a wider public health perspective is taken, the success of interventions and recommendations will be limited.

## Graphical abstract

**Figure 1:**
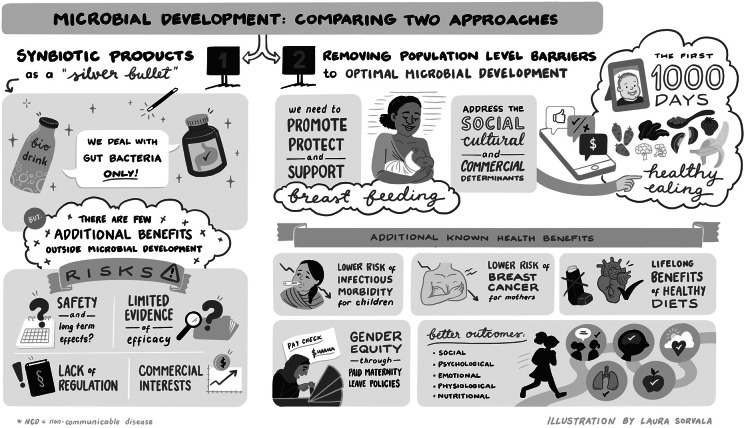

## Introduction

Non-communicable diseases (NCDs) present a serious global health challenge, accounting for over 70% of global deaths each year (Bennett et al., 2018). NCDs are often chronic and require years of medical management, creating immense strain on healthcare systems (Dietert & Dietert, 2015).

The United Nations Sustainable Development Goals (SDGs) set out clear targets to tackle growing cases of NCDs, with SDG Target 3.4 on Non-communicable Diseases and Mental Health encapsulating the aim of reducing premature deaths attributable to NCDs by one-third by 2030. Although some advances have been made, the rate of progress is slow, and many countries will not meet the 2030 target (Bennett et al., 2020).

Recent studies have identified that certain gut microbiome compositions may be associated with disease, including multiple NCDs like diabetes, cancer, asthma, and mental health conditions (Cong & Zhang, 2018; Simpson et al., 2021). Although this association has been established, the direction and causal strength of this relationship are not yet clear (Wade & Hall, 2019). Nonetheless, there is increasing interest in exploring the potential of the microbiome as a target for NCD prevention and reduction (Stinson, 2020).

Microbial colonisation during infancy plays an instrumental role in the development of the immune system, which then has sustained effects on health throughout life (Gensollen et al., 2016). As the majority of microbial development occurs within the first 1,000 days of life, this period presents a critical window in which modifiable exposures, such as nutrition, birth route, and antibiotic usage, could have a significant impact (Tamburini et al., 2016). It is also well-recognised that infants are highly susceptible to developmental programming effects during this stage of life (Robertson et al., 2019; Scott, 2020).

Within the academic literature, there is often a focus and priority placed on the science and pathophysiology surrounding novel strategies to tackle NCDs and a relative lack of emphasis on the application of this research in the real world. Without understanding the social, structural, and political context in which specific exposures can be targeted for intervention, it is difficult for recommendations to be implemented and well-meaning solutions to reach their full potential (Weiner et al., 2012). A broader public health perspective derived from Bronfenbrenner’s socioecological systems theory (Sallis et al., 2008) can shed light on the multiple levels at which factors influence microbial development in the 1,000 days, ranging from individual dietary behaviours and their interaction with the wider food system, to more upstream policy-level drivers of breastfeeding practices. Importantly, environmental interventions should precede educational strategies in order to reduce unrealistic expectations of individuals and their capacity to change their health behaviours (Sallis et al., 1998).

Although many modifiable factors can affect microbial development, nutritional factors are not easy to modify. In this narrative review, we explore the social, structural, and political barriers that may hinder healthy microbial development in the first 1,000 days in the context of current scientific knowledge, focusing solely on nutritional factors. We summarise the available evidence and explore obstacles to nutritional choices affecting microbial development, as well as unpack the rhetoric that healthy eating to develop a microbiome that supports optimum health is an individual choice.

## The gut microbiome and nutrition in the first 1,000 days

### What is the gut microbiome and why does it matter

The human body shelters a wide variety of microbial communities (Cong & Zhang, 2018; Valdes et al., 2018). The gastrointestinal (GI) or gut microbiota is the largest, most diverse, and complex, harbouring billions of bacteria (the predominant organisms), archaea, eukaryotes, and viruses (Ursell et al., 2014). The combined gut bacterial communities are referred to as the “gut microbiota,” while the totality of genes that compose the gut microbiota is referred to as the “gut microbiome” (Hou et al., 2022). This article refers specifically to the gut microbiome or microbiota, unless otherwise specified. The gut bacteria coexist and interact with the host, helping maintain homeostasis, both locally and systemically. They have a crucial role in food fermentation, pathogen defence, immune stimulation, and vitamin synthesis (Hillman et al., 2017). The composition of the early gut microbiota is typically assessed by analysing infant stool samples using sequencing technologies. The level of taxonomic detail researchers can obtain depends on the specific sequencing method used, with some techniques offering higher resolution than others (Petersen & Round, 2014; Franzosa et al., 2015; Young, 2017; Ruan et al., 2020; Stinson, 2020).

The initial gut microbiota colonisation starts at birth (Perez-Muñoz et al., 2017). The gut microbiota composition and assembly are highly dynamic during the neonatal period and infancy, stabilising to resemble the adult gut microbiota at 1–3 years of age (Bäckhed et al., 2015). Many different factors shape the gut microbiota in early life, including maternal microbiota composition (which, in turn, is greatly influenced by maternal diet), gestational age, mode of birth, antibiotic exposure, genetics, and infant feeding practices (Bäckhed et al., 2015; Shao et al., 2019). Delivery mode is the main driver of the initial gut microbiome composition. Babies born vaginally generally show microbiome compositions enriched with microbes of the genera Bifidobacterium, Escherichia, Bacteroides, and Parabacteroides (Bäckhed et al., 2015; Shao et al., 2019). Most of the maternal bacterial gut and vaginal microbiota strain transmissions during the neonatal period happened in babies born vaginally, while babies born by caesarean section show more microorganisms from the skin and oral microbiota and the surrounding environment, including potentially pathogenic bacteria such as microbes of the genera Enterococcus, Enterobacter, and Klebsiella (Bäckhed et al., 2015; Shao et al., 2019). The differences between delivery modes generally tend to diminish by 6–12 months of age as feeding patterns and solid food introduction become more influential (Bäckhed et al., 2015).

There is growing evidence that early gut microbiota composition is associated with later risk of NCDs, such as obesity, asthma, diabetes, and cardiovascular conditions (Sarkar et al., 2021). For instance, a longitudinal study found that infants with higher *Bifidobacterium* levels were more likely to maintain a healthy weight at age 7 years, whereas those with higher *Staphylococcus aureus* were more likely to become overweight (Kalliomäki et al., 2008). Other studies report inverse associations between Bifidobacterium abundance and markers like glucose intolerance, body mass index, and systemic inflammation (Bjursell et al., 2011; Conterno et al., 2011). In animal models, lower ratios of Firmicutes (i.e., Bacillota) (Parks et al., 2020) to Bacteroidetes and reduced butyrate-producing bacteria have been linked to diabetes development (Siljander et al., 2019).

Epidemiological studies further highlight associations between early-life exposures and NCDs. Prenatal undernutrition and low birth weight have been associated with higher risks of obesity, cardiovascular disease, and diabetes later in life (Barouki et al., 2012; Hanson & Gluckman, 2015). Similarly, maternal obesity and gestational diabetes are associated with long-term metabolic risks in both mother and child (Barouki et al., 2012). Breastfeeding, on the other hand, is consistently associated with lower rates of childhood obesity and adiposity (Ma et al., 2020). Despite limitations in the current research, evidence increasingly suggests a link between the early-life gut microbiome and the development of NCDs. This underscores the importance of identifying windows of opportunity for intervention, particularly through diet, one of the most modifiable factors influencing microbiome composition during the first 1,000 days of life. There are three distinct nutritional phases in the first 1,000 days of life in which dietary interventions and exposures can be implemented – (1) the maternal diet in the prenatal period, (2) early infancy when breastmilk is the only required source of nutrition, and (3) the complementary feeding period (Mameli et al., 2016). Within each period, numerous factors influence microbial composition (Tamburini et al., 2016; Stinson, 2020).

### Nutritional factors that affect microbial composition in pregnancy

The importance of nutrition during pregnancy has long been recognised, particularly by the Developmental Origins of Health and Disease Hypothesis, which proposes that in-utero environments influence developmental pathways and future infant health (Chu et al., 2016a,b; Stinson, 2020; Maher et al., 2023). Despite there being evidence that maternal nutrition in pregnancy affects future health outcomes of the infant, the exact mechanism through which this occurs has thus far remained vague (Brumana et al., 2017).

Maternal diet can have both positive and negative impacts on the infant’s microbiome. It has been demonstrated that gestational high-fat diets lead to a reduction of *Bacteroides* in the infant’s microbiome (Chu et al., 2016a,b). The reduction in *Bacteroides* species could impact the development of immunity and energy extraction of some polysaccharides (Chu et al., 2016a,b).

Conversely, diets rich in fruits, vegetables, and fibre have been associated with infant microbial compositions lower in unhealthy gut microbes, such as *Erysipelatoclostridium*, Betaproteobacteria, and Lachnospiraceae (Fan et al., 2021).

Further research is required in this area as significant gaps remain. Many studies fail to account for other variables in this time period, such as delivery mode, thus making it challenging to determine if maternal diet in isolation has a direct impact on infant microbial composition.

### Breastfeeding and the developing microbiome

After birth, colonisation of the infant microbiome continues and is highly influenced by human milk consumption, with breastfeeding recognised as one of the most significant factors affecting microbiome development in the first few years of life (Bäckhed et al., 2015; Stewart et al., 2018; Shao et al., 2019).

Breastfeeding shapes the early gut microbiota composition through different mechanisms. Through direct transfer of microbes, as well as nutrients that stimulate selective bacterial growth, including human milk oligosaccharides or casein glycol-macropeptides (Moossavi et al., 2018; Boudry et al., 2021).

Distinct differences have been identified between the microbiota of exclusively breastfed and exclusively formula-fed infants (Bäckhed et al., 2015). Despite the advances in formula milk composition, formula-fed infants still display noticeable microbial features, with an under-representation of *Lactobacillus*, which leads to an over-representation of other microorganisms such as *Clostridium difficile*, compared to breastfed infants (Azad et al., 2013; Bäckhed et al., 2015; Tanaka & Nakayama, 2017). Breastfed infants also seem to exhibit lower microbial diversity compared to formula-fed infants (Young & Schmidt, 2008; Azad et al., 2013; Davis et al., 2020).

### Complementary feeding

Complementary feeding marks the introduction of semi-solid and solid food into the infant’s diet, when breastmilk or formula milk no longer meets all their nutritional needs (Campoy et al., 2018). When solid foods are introduced into the diet, a large microbial transition occurs, causing a shift towards a more stable and adult-like microbiome (Laursen et al., 2016). Despite this shift in composition, it is the subsequent cessation of breastfeeding that seems to have a more pronounced effect on microbial development (Bäckhed et al., 2015).

In the complementary feeding phase, the microbial composition changes from being mainly characterised by lactobacilli, bifidobacteria, and enterobacteria to microbiota dominated by *Clostridium* spp. and *Bacteroides* spp. (Bergström et al., 2014). As complementary feeding continues, the differences between the microbiomes of breastfed and formula-fed infants decrease, disappearing by 2–3 years and stabilising to an adult-like phase (Bergström et al., 2014; Stinson, 2020).

There is some evidence that early food patterns and dietary habits in the early years of life can affect the gut microbiome composition (Laursen et al., 2016). However, there is a lack of high-quality studies in this phase of development as a result of diet in early childhood being difficult to measure accurately, and there are very few studies exploring the association between specific diets and gut microbiome composition in young children.

## Taking a public health nutrition perspective on the gut microbiome

The interest surrounding the microbiome has substantially increased in the last few years, and it is likely that nutritional factors during the first 1,000 days of life could influence microbial development and have significant impacts on health programming and future disease (Tamburini et al., 2016). However, there are limitations surrounding the current research, the largest of which is the lack of longitudinal evidence estimating causal effects of interventions in the first 1,000 days targeting the microbiome on microbial composition and risk of future disease. In addition, most studies regarding microbiome composition have been undertaken in high-income countries (HICs) (Sarkar et al., 2021). There is a gap in research on factors that may affect microbial composition in low- and middle-income countries (LMICs). Addressing limitations related to causal evidence on intervention effectiveness over the life course and estimating the strength of these relationships across global contexts would contribute to a much stronger evidence base. Despite these evidence gaps on the most effective interventions for optimal microbial development and limited insight into mechanistic variation across the globe, the underlying positive influence of breastfeeding and healthy diets in the 1,000 days on decreased risk of NCDs is applicable and relevant across contexts, forming a core area of importance for public health nutrition action in all countries. However, in parallel to resolving issues surrounding the research, it is important to pay close attention to population-level barriers that may hinder microbial development through effective public health nutrition strategies. Without addressing both issues, interventions will have a limited impact.

In the first 1,000 days of life, it is specifically important to target barriers surrounding breastfeeding and tackle the rhetoric on healthy maternal and child diets that ignores the social determinants of health, but instead places responsibility on individual women for children’s long-term health and development.

### Barriers and solutions to healthy eating and breastfeeding can be identified using the social-ecological model

When exploring barriers surrounding nutrition and breastfeeding, it is helpful to break them down using the social-ecological model (SEM). The SEM demonstrates interactions between individuals and their environments, and the influences that shape health behaviours (McLeroy et al., 1988).

At an individual level, barriers to a healthy microbiome through diet or breastfeeding include limited knowledge, restricted income, and time constraints (Kavle & Landry, 2018; Kay et al., 2025). Integrating nutritional education and education on breastfeeding into antenatal appointments can help reduce some of these barriers (Olloqui-Mundet et al., 2024).

At an interpersonal level, support from family and friends is key to making positive nutritional choices. Lack of support and detrimental cultural practices can cause significant barriers to healthy eating and breastfeeding (Snyder et al., 2021; Neven et al., 2022). Mothers who have a supportive partner are more likely to breastfeed, as well as women who have family members who have breastfed (Lok et al., 2017; Gebrekidan et al., 2020).

At a community level, systemic issues lead to barriers. In rural areas, difficult access to fresh food can force individuals to opt for highly processed, unhealthy options (Whelan et al., 2018). Without adequate support and sufficient infrastructure, it is hard for families to make healthy nutritional choices. Community-based support groups, positive social norms, and urban gardens can all help promote healthy behaviours and breastfeeding (Shakya et al., 2017; Garcia et al., 2018).

At the policy level, the absence of robust legislation and public health initiatives causes significant barriers. Lack of legislation, or enforcement of legislation, regulating corporate actors in the formula milk industry has had detrimental effects on breastfeeding rates globally (Baker et al., 2021; Lutter et al., 2022). Alongside this, policies designed to protect and encourage breastfeeding are lacking, including failure to ensure adequate maternity leave, separating infants and mothers at birth, and failure to address stigma associated with breastfeeding in public (Rollins et al., 2016; Grant et al., 2022). Healthy food is often unaffordable. Although some governments have imposed taxes on high-sugar foods, very few have introduced subsidies to make nutritious foods more accessible (Blakely et al., 2020).

While individual choices influence dietary decisions and breastfeeding, the SEM demonstrates that these behaviours are shaped by interpersonal, community, and policy-level factors. Solutions to these barriers can be found at every level, but often the most impactful and lasting solutions come from a policy level. Recognising this shifts the narrative that places full responsibility for healthy choices on individuals and allows for a more equitable approach to public health.

### To boost microbial development at the population level, we must do more to protect, promote, and support breastfeeding

One of the most effective ways to increase healthy microbial development is breastfeeding (Stewart et al., 2018). There are many well-known health benefits associated with breastfeeding, including a lowered risk of infectious morbidity and mortality in children and a reduced risk of breast cancer in mothers (Victora et al., 2016). Despite this knowledge, breastfeeding rates are low in many parts of the world (UNICEF Data, 2018). In LMICs, 1 in 25 infants are never breastfed, but in HICs this rises to 1 in 5 children not receiving any breastmilk at all (UNICEF Data, 2018).

The determinants of breastfeeding are complex and multifactorial, ranging from social and cultural attitudes, unsupportive environments, and the commercial interests of the formula milk industry (Rollins et al., 2016).

Many commercial products are known for increasing the risk of developing NCDs, but the goods involved are highly profitable for companies and highly convenient for consumers (Horton et al., 2021). The breastmilk substitute industry is no exception to this. It is estimated to be worth US$70 billion (van Tulleken et al., 2020). Commercial marketing of formula milk negatively impacts breastfeeding rates (Rollins et al., 2016). Despite legislation published by the World Health Organization (WHO) attempting to outlaw advertising and promotion of formula milk, consumption of breastmilk substitutes remains widespread in HICs and is rising rapidly in the rest of the world, while some countries, including the United States, have not adopted the legislation into national guidelines (Soldavini & Taillie, 2017; Hastings et al., 2020).

Alongside commercial marketing, barriers to breastfeeding are also seen within healthcare systems and workplaces (Tomori, 2022). An important barrier to address is the environment created in hospitals to successfully initiate and establish breastfeeding. Early initiation and exclusive breastfeeding are often key to successful breastfeeding in line with public health recommendations, and require the mother and child to be within continuous reach of each other (Tomori, 2022). The Baby Friendly Hospital Initiative, based on the Ten Steps for Successful Breastfeeding, was created by the WHO in conjunction with the United Nations Children’s Fund (UNICEF) to try and improve breastfeeding rates and duration in hospitals (Fallon et al., 2019). This guidance educates healthcare staff and parents about breastfeeding and ensures mothers and infants are able to remain together within the hospital environment (WHO & UNICEF, 2009).

As the post-partum period continues, more barriers to breastfeeding become apparent. It has been found that paid maternity leave is a key determinant of breastfeeding. Maternity leave encourages both breastfeeding initiation and duration (Lauzon-Guillain et al., 2019).

To improve breastfeeding rates, support and intervention will be required at many levels – from personal and social influences to legal and political legislation that promotes and enables breastfeeding (Rollins et al., 2016).

If we believe that breastfeeding is important for microbial development, then globally, the current state of low breastfeeding rates will be a barrier to leveraging its potential to address NCDs through the pathway of microbial development, as will the commercial interests in the sphere of infant and young child feeding.

### The healthy diet and “breast is best” rhetoric places too much emphasis on individual choice

The wider discourse that nutritional decisions in the first 1,000 days are solely dictated by women’s individual choice and preference needs to be challenged.

There are many determinants surrounding food choice, including environmental, cultural, familial, and genetic factors (Leng et al., 2017). Global and national policymaking often overlooks many of these determinants. Little regard is paid to socio-economic factors, despite the evidence showing that higher quality diets are generally consumed by people of a higher economic status and lower quality diets tend to be consumed by less affluent populations (Darmon & Drewnowski, 2008).

Oversimplification of the “healthy eating” rhetoric that places all responsibility on individual choice and ignores the social determinants of health has thus far failed to stem the rising burden of NCDs (Kriznik et al., 2018). Instead, a more integrated approach that considers structural barriers alongside the social and commercial determinants of health must be applied.

We must also examine the pressure and responsibility that is placed on women and mothers to provide good-quality infant nutrition and exclusively breastfeed for 6 months. We live in a world that sexualises and stigmatises women’s bodies while simultaneously shaming them for not breastfeeding. When women are unable or choose not to breastfeed, there is often a narrative of blame, which further places responsibility on the individual woman rather than examining the structural, social, and political barriers that may have contributed to the outcome (Tomori, 2022).

### Symbiotic products – another silver bullet that misses the mark?

Like most global public health nutrition issues, the discourse on healthy microbial development in the first 1,000 days displays a predilection for fast fixes. “Silver bullet” solutions, such as pro- and prebiotics, claim to be the key to a healthy microbiome. The symbiotic industry, which includes probiotics and prebiotics products, is estimated to be a multi-billion-dollar market (Quin et al., 2018). While consumers generally view these products as beneficial for their health, there is often a lack of knowledge on how the products work, and evidence on safety and efficacy is patchy (Cunningham et al., 2021). These issues come as a consequence of the symbiotic industry being largely unregulated and governance varying between different regions and countries (de Simone, 2019). There have been relatively few studies that evaluate the use of symbiotic products for developing and maintaining the early microbiome. Among the few studies that have been done, the results are varied, and many call for further studies into the long-term effects and safety of these products (Quin et al., 2018; Westaway et al., 2022).

While these commercial symbiotic products might have some benefits for the microbiome, they do not match the additional benefits that breastfeeding and healthy diets confer in ways unrelated to the microbiome. The pathways linking breastfeeding and healthy eating to lower NCD risk can thus be split into two – one mediated by microbial development and a separate pathway encapsulating all other benefits related to social, psychological, emotional, physiological, and nutritional processes. The impact of symbiotics on NCDs will, therefore, be smaller than the total effects of the more holistic benefits of breastfeeding and healthy diets. If breastfeeding and healthy diets can do the same job as symbiotics and much more besides, why not develop interventions, policies, and support for these instead of hoping that yet another silver bullet in the form of symbiotics will solve a global public health crisis?

## Conclusion

It is likely that promoting healthy microbial development through informed and accessible nutritional choices in the first 1,000 days of life could have a significant impact on the health of future generations and reduce the global NCD burden.

As more evidence emerges to support the association between nutritional factors and healthy microbial development, it will be important to acknowledge and address the limitations surrounding the current research. However, it is imperative that we also anticipate and tackle population-level barriers that may hinder healthy microbial development, particularly breastfeeding and healthy eating. Until this is done, it will be difficult to harness the full potential of healthy microbial development as a strategy to reduce the future risk of NCDs.

## Data Availability

This review article did not generate any primary data and used information already available from published sources.
